# Modulation of Primary Immune Response by Different Vaccine Adjuvants

**DOI:** 10.3389/fimmu.2016.00427

**Published:** 2016-10-17

**Authors:** Annalisa Ciabattini, Elena Pettini, Fabio Fiorino, Gabiria Pastore, Peter Andersen, Gianni Pozzi, Donata Medaglini

**Affiliations:** ^1^Laboratory of Molecular Microbiology and Biotechnology, Department of Medical Biotechnologies, University of Siena, Siena, Italy; ^2^Department of Infectious Disease Immunology, Statens Serum Institut, Copenhagen, Denmark

**Keywords:** adjuvants, CD4^+^ T cell priming, B cell priming, MHC class II tetramers

## Abstract

Adjuvants contribute to enhancing and shaping the vaccine immune response through different modes of action. Here early biomarkers of adjuvanticity after primary immunization were investigated using four different adjuvants combined with the chimeric tuberculosis vaccine antigen H56. C57BL/6 mice were immunized by the subcutaneous route with different vaccine formulations, and the modulation of primary CD4^+^ T cell and B cell responses was assessed within draining lymph nodes, blood, and spleen, 7 and 12 days after priming. Vaccine formulations containing the liposome system CAF01 or a squalene-based oil-in-water emulsion (o/w squalene), but not aluminum hydroxide (alum) or CpG ODN 1826, elicited a significant primary antigen-specific CD4^+^ T cell response compared to antigen alone, 7 days after immunization. The effector function of activated CD4^+^ T cells was skewed toward a Th1/Th17 response by CAF01, while a Th1/Th2 response was elicited by o/w squalene. Differentiation of B cells in short-lived plasma cells, and subsequent early H56-specific IgG secretion, was observed in mice immunized with o/w squalene or CpG adjuvants. Tested adjuvants promoted the germinal center reaction with different magnitude. These results show that the immunological activity of different adjuvants can be characterized by profiling early immunization biomarkers after primary immunization. These data and this approach could give an important contribution to the rational development of heterologous prime–boost vaccine immunization protocols.

## Introduction

Adjuvants are key components in the vaccine formulations since they provide the necessary help for enhancing and shaping the vaccine immune responses. Although vaccine adjuvant research was at first based on empirical approaches, the new insights into the immunological mechanisms involved in the vaccination response have now allowed the improvement of knowledge in the mode of action of various compounds. This is particularly due to the intriguing role of the innate response, together with the development of novel technical tools. Among the priorities on the innovations to boost research in the field of vaccine is the generation of a toolbox of adjuvants, with a well-defined profile to shape the immune response, which can be applied to vaccines against diverse pathogens (www.iprove-roadmap.eu) (1). New adjuvants are also needed to improve existing vaccines in different population groups for which the activity of current adjuvants may differ. These include the elderly, infants, and chronically infected subjects that mount a suboptimal immune response to vaccination (2–4). Profiling the mode of action of different adjuvants is of critical importance for the rationale design of vaccination strategies, based on heterologous combinations of vaccine formulations for priming and boosting (5–8).

According to their mechanism of action, adjuvants are commonly classified as immunomodulatory molecules, delivery systems, or a combination of both (9). Immunomodulatory molecules mainly activate the innate immune receptors, such as TLRs, NOD-like receptors, C-type lectins, and RIG-I-like receptors, generating signals that determine the proper activation of downstream adaptive immune responses and cells homing. The nature of the initial “danger” signals perceived by innate immune cells can dictate the type, quality, and magnitude of the adaptive immune response. This offers unique opportunities to custom tailor new classes of adjuvants to generate desired types of immune response. Delivery systems – such as mineral salts, liposomes, microparticles, saponins, and emulsions – improve the delivery and presentation of the vaccine to the immune system (9). Combination adjuvants include more components that act synergistically by activating a variety of immune mechanisms (10).

The immunological signature of adjuvants is generally established at the end of immunization schedules including booster immunizations (11, 12), and secondary immune responses are analyzed to compare their adjuvanticity or describe novel potential adjuvant molecules. Within the ADITEC project (13), a unique effort has been conducted to compare head to head the mechanism of action and efficacy of five different adjuvants, combined with vaccine antigens from *Mycobacterium tuberculosis*, influenza, and Chlamydia (14). Nevertheless, when a vaccine formulation is injected into the host, the first influence on the magnitude, type, and quality of the downstream immune response, including the generation of memory, is elicited at the priming event. For this reason, the characterization of the adjuvant properties of a vaccine formulation at an early time point during the primary response is of critical importance for the rationale design of vaccination strategies and optimal prime–boost combinations.

The primary response to vaccination takes place locally, within the draining lymph nodes, where the antigen-presenting cells present the vaccine antigen to specific CD4^+^ T cells that proliferate and differentiate into various functionally defined subset effector cells (15). One of these subtypes is T follicular helper (Tfh) cells that relocate to B–T cell borders and interfollicular regions (16, 17). They are specialized in regulating multiple stages of antigen-specific B-cell immunity and triggering their differentiation into long-lived plasma cells (PCs) or memory B cells. In the extra-follicular reaction, some antigen-primed B cells, after cognate contact of Tfh cells, undergo a process of rapid differentiation in short-lived PC. As a result, they produce low affinity antibodies, such as IgM and IgG, which appear in serum at low concentration a few days after immunization, and undergo apoptosis after a few days of intensive antibody secretion (16, 18). Interaction of Tfh cells with B cells also drives the germinal center (GC) reaction, a dynamic microanatomical structure that supports the generation of B-cell activation, antibody class switch recombination, and affinity maturation (17, 19). T-cell priming is therefore an essential event for the induction of the adaptive immune response to vaccination, and it represents a key step in the vaccination process due to the close relationship with long-term humoral immunity and protective antibodies (18).

T-cell priming is influenced by the type of vaccine formulation (antigen, adjuvant, delivery system), the dose, and the route of administration (20). We have recently demonstrated that the subcutaneous (SC) route was particularly efficient in priming antigen-specific CD4^+^ T cells capable of responding to booster immunization, whereas the T-cell response induced after nasal priming was poorly responsive to recall immunization (7). The modulation of CD4^+^ T cell priming has been characterized for bacterial delivery systems (21–23) and adjuvants, such as alum (24), lipopolysaccharide (25), or its derivative-like monophosphoril lipid A (26), cholera toxin (27), or its B subunit (CTB) (28, 29), CpG ODN (30), and the liposomal system CAF01 (7, 31). Different adjuvants have demonstrated the ability to induce Tfh cell differentiation (31–33). We have also exploited mathematical models as a tool to estimate *in vivo* the probability of antigen-specific CD4^+^ T cell expansion and dissemination upon immunization with adjuvanted vaccine formulations (34, 35).

In the present manuscript, in order to define early biomarkers of adjuvanticity, we have characterized the primary CD4^+^ T and B cell immune responses specific for the chimeric tuberculosis vaccine antigen H56 (36), elicited by four different adjuvants, alum, a squalene-based oil-in-water emulsion, CpG ODN 1826 (37), or the liposome system CAF01 (38). Our results show how different adjuvants modulate the acquired immune response to the vaccine antigen since the primary immunization, and highlight CD4^+^ T and B cell priming events as critical early biomarkers of adjuvanticity of different classes of molecules.

## Materials and Methods

### Mice

Seven-week-old female C57BL/6 mice, purchased from Charles River (Lecco, Italy) were housed under specific pathogen-free conditions in the animal facility of the Laboratory of Molecular Microbiology and Biotechnology (LA.M.M.B.), Department of Medical Biotechnologies at University of Siena, and treated according to national guidelines (Decreto Legislativo 26/2014). All animal studies were approved by the Italian Ministry of Health with authorization n° 1004/2015-PR on 22 September, 2015.

### Adjuvants and Immunizations

CAF01 [250 μg dimethyldioctadecylammonium (DDA) and 50 μg trehalose dibehenate (TDB)/mouse; Statens Serum Institut, Denmark], CpG ODN 1826 (hereafter CpG, 20 μg/mouse; Eurofins MWG Operon, Germany), AddaVax squalene-based oil-in-water adjuvant [hereafter o/w squalene, 50 μl/mouse, sorbitan trioleate (0.5% w/v) in squalene oil (5% v/v), and Tween 80 (0.5% w/v) in sodium citrate buffer (10 mM, pH 6.5), Invivogen, USA], or aluminum hydroxide (hereafter alum, 0.5 mg/mouse; 2% alhydrogel, Brenntag Biosector, Denmark) were mixed with H56 antigen (2 μg/mouse; Statens Serum Institut, Denmark). Vaccine formulations were subcutaneously injected, at the base of the tail, in a volume of 150 μl/mouse of 10 mM Tris for CAF01, of 100 μl/mouse of PBS for CpG, and o/w squalene adjuvants or distilled water for alum. Control mice received 2 μg of H56 alone in 100 μl/mouse of PBS, while naïve mice were left as negative control. Mice were immunized at day 0 and sacrificed on days 7 and 12.

### Sample Collection and Cell Preparation

Draining lymph nodes (sub iliac, medial, and external) and spleens were collected 7 and 12 days after priming. Samples were mashed onto 70-μm nylon screens (Sefar Italia, Italy) and washed two times in complete medium [RPMI medium (Lonza, Belgium) supplemented with 100 U/ml penicillin/streptomycin and 10% fetal bovine serum (Gibco, USA)]. Samples were treated with red blood cells lysis buffer according to the manufacturer’s instruction (eBioscience, CA, USA). Blood samples were taken on days 7 and 12. Samples were incubated for 30 min at 37°C, centrifuged at 1200 × *g* at 4°C for 10 min, and sera were then collected and stored at −80°C until analysis.

### Flow Cytometric Analysis and Intracellular Cytokine Staining

Samples were incubated for 30 min at 4°C in Fc-blocking solution [complete medium with 5 μg/ml of CD16/CD32 mAb (clone 93; eBioscience, CA, USA)]. To evaluate tetramer-specific CD4^+^ T cells and follicular T cells in draining lymph nodes, cells were stained for 1 h at RT with PE-conjugated I-A(b) *M. tuberculosis* Ag85B precursor 280–294 (FQDAYNAAGGHNAVF) tetramer (kindly provided by NIH MHC Tetramer Core Facility, Emory University, Atlanta, GA, USA) together with BV650-conjugated anti-CXCR5 (clone 2G8, BD Biosciences, USA). Cells were washed and surface stained with HV500-conjugated anti-CD4 (clone RM4-5; BD Biosciences), APC-conjugated anti-CD44 (clone IM-7; Biolegend), BV786-conjugated anti-CD273 (PD-1, clone TY25; BD Biosciences). GC B cells were detected by staining with PE-conjugated anti-CD45R (anti-B220, clone RA3-6B2; BD Biosciences), BV421-conjugated anti-GL-7 (clone GL-7; BD Biosciences), PerCP e-Fluor 710-conjugated anti-CD95 (clone 15A7; eBioscience). PCs were evaluated by staining with AF700-conjugated anti-CD45R (B220, clone 15A7; eBioscience), BV605-conjugated anti-IgD (clone 11-26C.2A; BD Biosciences), BV421-conjugated anti-CD138 (clone 281-2; BD Biosciences). Samples were labeled with LIVE/DEAD Fixable Near IR Dead Cell Stain Kit according to the manufacturer’s instruction (Invitrogen, USA). Intracellular staining for PE-CF594-conjugated anti-Bcl-6 (clone K112-91, BD Biosciences) was performed using the FoxP3 staining buffer set (eBioscience) according to the manufacturer’s instruction. Intracellular cytokine production was assessed on splenocytes cultured for 6 h in the presence of anti-CD28, anti-CD49d (both 2 μg/ml, eBioscience), and H56 protein (2 μg/ml), or stimulated with PMA and ionomycin calcium salt (50 ng/ml and 1 μM, respectively, Sigma-Aldrich) for positive control. Brefeldin A (BFA, 5 μg/ml, Sigma-Aldrich) and monensin solution (eBioscience) were added for the last 4 h of incubation. Cells were washed twice in PBS and then labeled with LIVE/DEAD Fixable Yellow Dead Cell Stain Kit according to the manufacturer’s instruction (Invitrogen, USA). Fixation and permeabilization were performed using BD Cytofix/Cytoperm kit according to the manufacturer’s instruction (BD Biosciences) before Fc-blocking and staining with HV500-conjugated anti-CD4 (clone RM4-5; BD Biosciences), APC-conjugated anti-CD44 (clone IM-7; Biolegend), PerCP Cy5.5-conjugated anti-IFN-γ (clone XMG1.2; BD Biosciences), PE-conjugated anti-IL-2 (clone JES6-5H4; BD Biosciences), AF700-conjugated anti-TNF-α (clone MP6-XT22; BD Biosciences), APC-conjugated anti-IL-17A (clone eBio17B7; eBioscience), AF488-conjugated anti-IL-4 (clone 11B11; eBioscience), and AF488-conjugated anti-IL-13 (clone eBio13A; eBioscience). Antibodies and tetramer were titrated for optimal dilution. About 5–10 × 10^5^ cells were stored for each sample acquired on LSR-II flow cytometer (BD Biosciences). Data analysis was performed using FlowJo (TreeStar, USA).

### Multiplex Cytokine Assay

IL-1α, IL-1β, IL-2, IL-3, IL-4, IL-5, IL-6, IL-10, IL-12p40, IL-12p70, IL-13, IL-17A, eotaxin, G-CSF, GM-CSF, IFN-γ, KC, MCP-1, MIP-1α, MIP-1β, RANTES, and TNF-α production were assessed in culture supernatants of restimulated splenocytes by Luminex immunoassay (BioRad, USA). Splenocytes were cultured with 2 μg/ml of H56 in complete medium for 72 h at 37°C in 5% CO_2_, supernatants were then collected, and stored at −80°C. Analytes were detected using the multiplex cytokine immunoassay (Bio-Plex, BioRad) following the manufacturer’s protocol and analyzed by Bio-Plex Magpix Multiplex Reader (BioRad). Cytokine concentrations were expressed as picograms per milliliter and were calculated based on standard curve data using Bio-Plex Manager 6.1.

### Enzyme-Linked Immunosorbent Assay

Serum H56-specific IgG, IgG1, and IgG2c were determined by enzyme-linked immunosorbent assay (ELISA). Flat-bottomed Maxisorp microtiter plates (Nunc, Denmark) were coated with H56 (0.5 μg/ml) for 3 h at 37°C and overnight at 4°C in a volume of 100 μl/well. Plates were washed and blocked with 200 μl/well of PBS with 1% BSA (Sigma-Aldrich) for 2 h at 37°C. Serum samples were added and titrated in twofold dilution in duplicate in PBS with 0.05% Tween 20 and 0.1% BSA (diluent buffer) in 100 μl/well. After incubation for 2 h at 37°C, samples were incubated with the alkaline phosphatase-conjugate goat anti-mouse IgG, IgG1, and IgG2c (each diluted 1:1000, Southern Biotechnology, USA) for 2 h at 37°C in 100 μl/well and developed by adding 1 mg/ml of alkaline phosphatase substrate (Sigma-Aldrich) in 200 μl/well. The optical density was recorded using Multiskan FC Microplate Photometer (Thermo Scientific). Antibody titers were expressed as the reciprocal of the highest dilution with an OD value ≥0.2, after background subtraction.

### Statistical Analysis

Mann–Whitney test with Bonferroni correction for multiple pairwise comparisons was used for assessing statistical difference between each group immunized with adjuvant and the H56-immunized group. The Kruskal–Wallis test, followed by Dunn’s post test for multiple comparisons, was used to assess statistical difference between groups immunized with different adjuvants A *P* value ≤ 0.05 was considered significant. Statistical analysis was performed using Graph Pad Prism version 6 (GraphPad Software, CA, USA).

## Results

The chimeric tuberculosis vaccine antigen H56 was combined with an o/w squalene-based emulsion, the liposome system CAF01, alum, or CpG and parenterally administered to mice in order to identify early biomarkers of adjuvanticity. The activity of the different adjuvants when combined with H56 was assessed by analyzing the primary T CD4^+^ and B cell responses within the local draining lymph nodes, blood, and spleen. Primary T-cell response was characterized in terms of antigen-specific CD4^+^ T cell expansion and cytokine secretion, while the mechanisms through which different adjuvants enhance B cell response were investigated by assessing PCs generation within draining lymph nodes and IgG antibodies release in blood, as well as analyzing the GC reaction within draining lymph nodes.

### Primary Ag-Specific CD4^+^ T Cell Expansion and Effector Function

The induction of antigen-specific CD4^+^ T cell expansion into the iliac draining lymph nodes was assessed 7 days after SC immunization with the H56 vaccine antigen alone or combined with the different adjuvants. CD4^+^ T cells specific for the immunodominant epitope of Ag85B, that is part of the chimeric H56 protein, were identified using Ag85B_280–294_-complexed MHC class II tetramers. Staining specificity was determined using a control tetramer complexed with an unrelated antigen, which showed a level of staining below 0.02% (data not shown).

**Figure 1:**
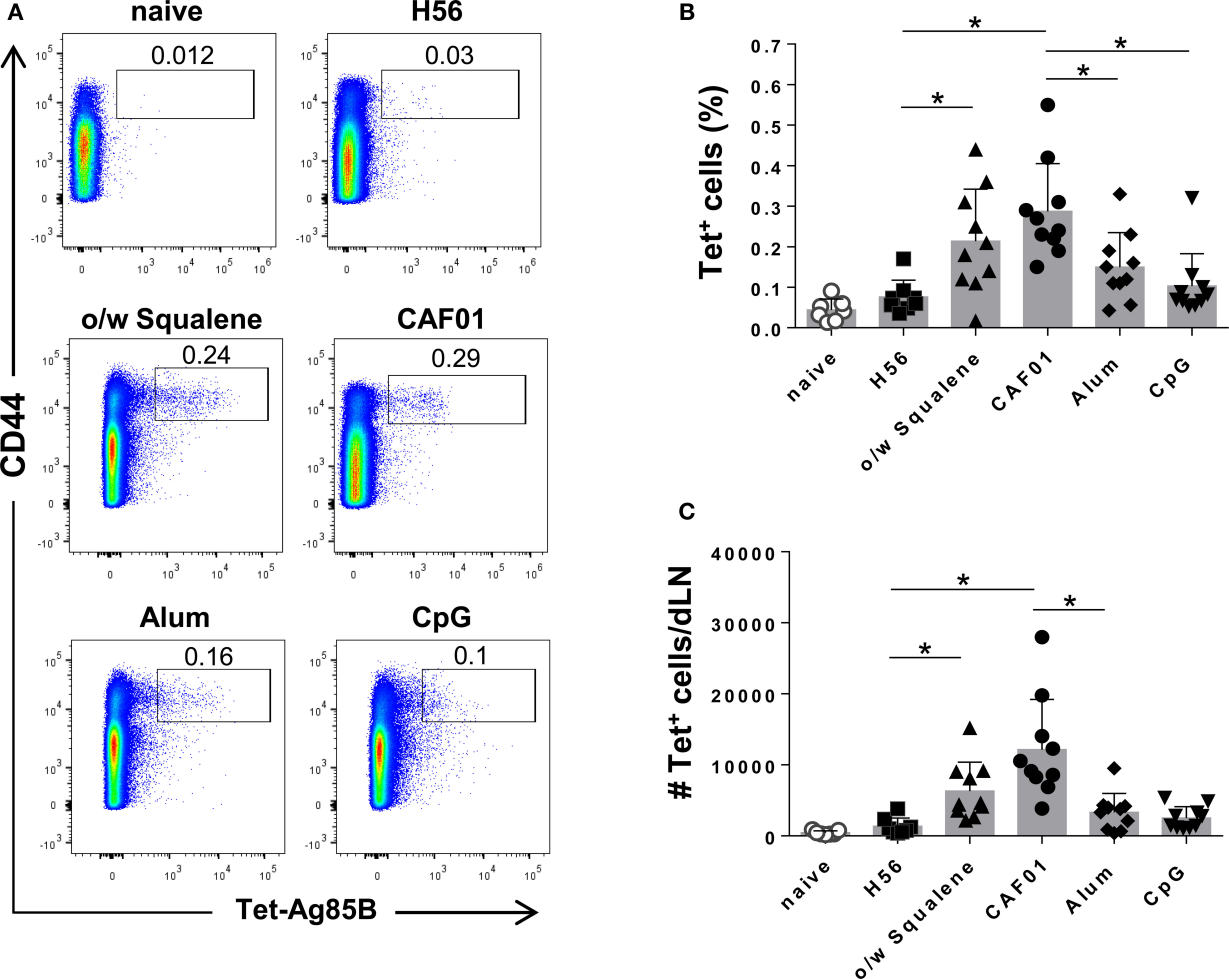
**Induction of Ag85B-specific CD4^+^ T cells**. C57BL/6 mice were subcutaneously immunized with H56 alone (H56) or combined with different adjuvants (o/w squalene, CAF01, alum, and CpG), and lymph nodes draining the site of immunization (dLN) were collected 7 days after immunization. Ag-specific T cells were identified by staining with Ag85B-specific MHC class II tetramers (Tet-Ag85B). **(A)** Tetramer^+^ T cells, detected as CD44^high^ Tet-Ag85B^+^ cells, gated on live CD4^+^ lymphocytes, are shown from a single animal representative of the group. **(B,C)** Frequencies of Tetramer^+^ CD44^+^ T cells, with respect to CD4^+^ cells **(B)** and absolute numbers of Tetramer^+^ CD44^+^ T cells per dLN **(C)** elicited by different vaccine formulations, reported as mean ± SD of 8–10 mice per group, from 2 independent experiments. Mann–Whitney test with Bonferroni correction for multiple pairwise comparisons was used for assessing statistical difference between each group immunized with adjuvant and the H56-immunized group. Kruskal–Wallis test, followed by Dunn’s post test for multiple comparisons, was used to assess the statistical difference between groups immunized with different adjuvants (*P ≤ 0.05).

Representative dot plots showing the frequencies of tetramer-positive (Tet^+^) T cells elicited by the different vaccine formulations are shown in Figure 1A. Priming with o/w squalene and CAF01 elicited a significant increase of Tet^+^ T cells, both in terms of frequency with respect to total CD4^+^ T cells and absolute number, compared to the group immunized with H56 antigen alone (Figures 1B,C), while vaccine formulations containing alum and CpG did not elicit a significant primary T helper response (Figures 1B,C). Comparison between different adjuvants indicated that immunization with CAF01 elicited a significant higher Ag-specific CD4^+^ T cell response compared to CpG and alum (Figure 1B). The analysis of percentage of Tet^+^ T cells elicited by the different vaccine formulations, repeated 12 days after priming, showed reduced frequencies in all groups (data not shown), suggesting that day 7 is a better time point for the analysis of antigen-specific T cell expansion in draining lymph nodes.

**Figure 2:**
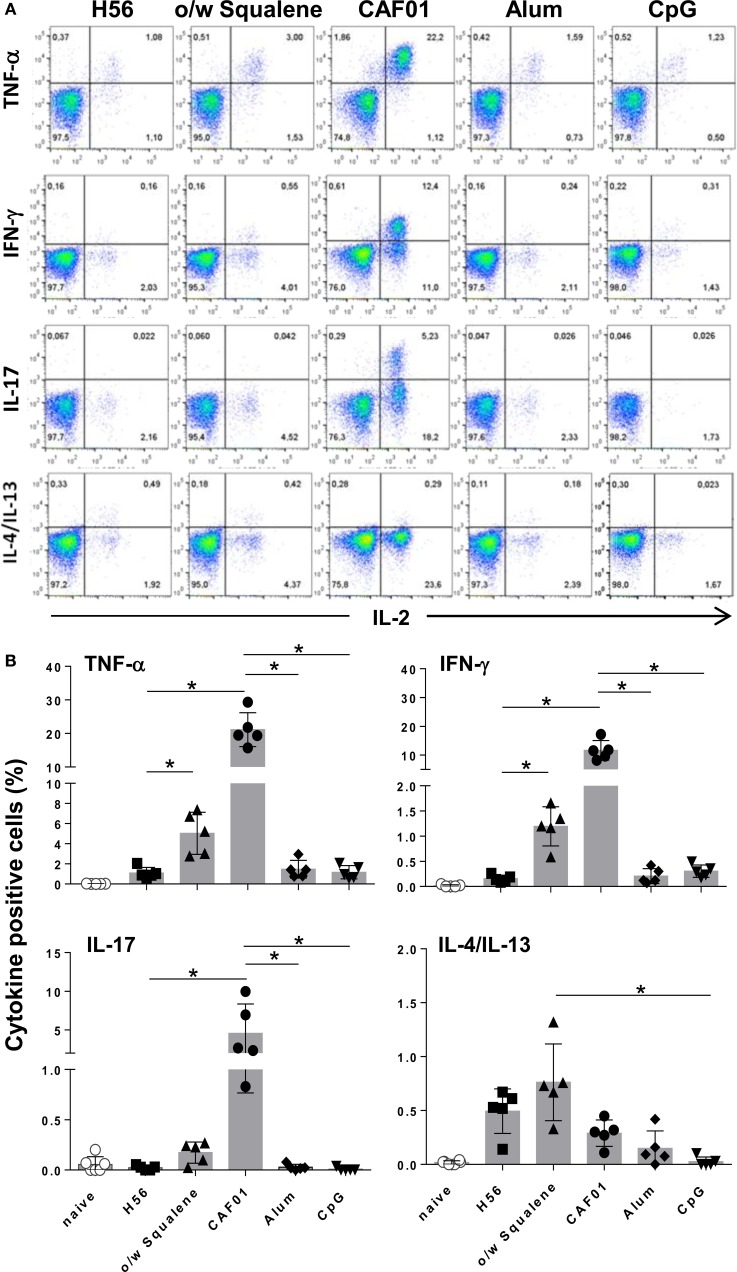
**Intracellular cytokines production**. C57BL/6 mice were subcutaneously immunized with H56 alone (H56) or combined with different adjuvants (o/w squalene, CAF01, alum, and CpG), and spleens were collected 7 days after immunization. Splenocytes were cultured for 6 h in the presence of anti-CD28, anti-CD49d, and H56 protein. **(A)** Dot plots showing the production of TNF-α, IFN-γ, IL-17, IL-4/IL-13 versus IL-2 assessed on live CD4^+^ CD44^+^ lymphocytes in each group. **(B)** Percentages of T cells positive for both IL-2 and the indicated cytokines, with respect to total CD4^+^ CD44^+^ cells, elicited by different vaccine formulations. Data are reported as mean ± SD of five mice per group. Mann–Whitney test with Bonferroni correction for multiple pairwise comparisons was used for assessing statistical difference between each group immunized with adjuvant and the H56-immunized group. Kruskal–Wallis test, followed by Dunn’s post test for multiple comparisons, was used to assess the statistical difference between groups immunized with different adjuvants (*P ≤ 0.05).

Since activated T helper cells exit the lymph nodes to recirculate, the effector function of H56-specific CD4^+^ T cells was assessed in splenocytes by flow cytometric analysis of intracellular cytokine production elicited by antigen restimulation. Figure 2A reports representative dot plots of the different groups showing the frequencies of H56-specific CD4^+^ T cells producing TNF-α, IFN-γ, IL-17, or IL-4/IL-13 cytokines versus IL-2, a cytokine indicative of the proliferative response and activation program of antigen-specific T cells. In all groups, IL-2 was always co-expressed with one of the other cytokines especially with TNF-α, as clearly shown in Figure 2A. CAF01 induced the overall highest recall response upon antigen restimulation, with significant percentages of cells expressing TNF-α/IL-2 (21% of CD4^+^ CD44^+^ T cells), IFN-γ/IL-2 (12%), and IL-17/IL-2 (4%) with respect to H56-immunized mice and also toward CpG and alum (Figure 2B; *P* ≤ 0.05). Vaccine formulation containing o/w squalene induced also a significant increase of TNF-α/IL-2 (5%) and IFN-γ/IL-2 (1.2%) producing cells compared to H56-immunized mice, together with a subpopulation of cells releasing IL-4/IL-13 (0.8%), indicative of a mixed Th1/Th2 response (Figure 2B). Cytokine production was not observed in groups immunized with alum and CpG adjuvants, while administration of the H56 antigen alone stimulated the release of IL-4/IL-13 (Figure 2B).

**Figure 3:**
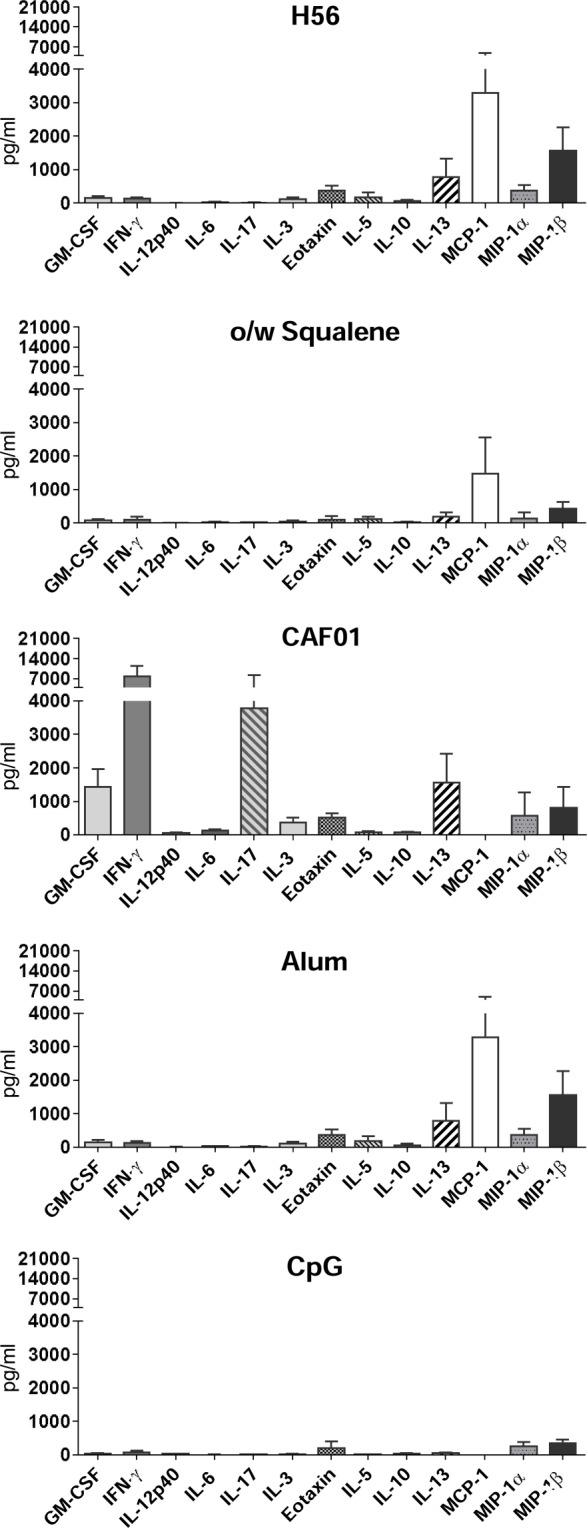
**Cytokine/chemokine production in splenocyte culture supernatants**. C57BL/6 mice were subcutaneously immunized with H56 alone (H56) or combined with different adjuvants (o/w squalene, CAF01, alum, and CpG), and spleens were collected 7 days after immunization. Cytokine secretion was detected upon 3 days of H56 antigen restimulation by Luminex immunoassay. Values, expressed as picograms per milliliter, are reported as the arithmetic mean ± SD of H56-stimulated *minus* the respective unstimulated samples, of five animals per group.

The levels of secreted cytokines upon a longer period of antigen restimulation (72 h) were measured by multiplex assay, including 22 different cytokines/chemokines. Concentrations of all soluble factors, measured in each tested group, are reported in Table S1 in Supplementary Material. In Table 1 and Figure 3 are reported the concentration and the fold change of the 13 cytokines and chemokines that were modulated by the tested adjuvants with at least a threefold change with respect to antigen alone.

**Table 1:** **Ratio of cytokine or chemokine production between formulations including adjuvants and antigen alone**.

	o/w squalene	CAF01	Alum	CpG
GM-CSF	0.5	11	1	0.4
IFN-γ	2	168	2.8	1.5
IL-12p40	0.5	3	1.4	2
Il-6	1.2	6	1.1	0.2
IL-17A	2.7	604	2.1	1.7
IL-3	0.4	3.6	1.1	0.17
Eotaxin	0.3	1.7	1.2	1.2
IL-5	0.1	0.07	0.2	0.01
IL-10	0.2	0.8	0.6	0.3
IL-13	0.1	0.8	0.4	0.03
MCP-1	0.4	0.17	1.1	0.16
MIP-1α	0.3	0.9	0.6	0.4
MIP-1β	0.2	0.4	0.7	0.2

*The H56-specific cytokine and chemokine production was assessed in mice immunized with formulations containing adjuvants (o/w squalene, CAF01, alum, or CpG) or antigen H56 alone, by Luminex immunoassay. The ratio between the cytokine or chemokine concentrations detected in samples from mice immunized with adjuvants respect to antigen alone is reported. Filled squares indicate an increase (dark gray) or decrease (light gray) of at least threefolds*.

The strongest modulatory effect was observed in the presence of CAF01, which induced a high increase of the cytokines GM-CSF, IFN-γ, IL-12p40, IL-6, IL-17A, and IL-3 with respect to the antigen alone (Figure 3; Table 1). O/w squalene and CpG were similar in eliciting a decrease of IL-5, IL-10, IL-13, and MIP-1β (Figure 3; Table 1). Alum showed a poor modulatory effect with respect to antigen alone, except for IL-5 that was the only cytokine down-modulated by all the adjuvants tested (Table 1).

In conclusion, o/w squalene and CAF01 promoted the antigen-specific CD4^+^ T cell proliferative response, which shifted to a Th1/Th17 response in the presence of CAF01, and to a mixed Th1/Th2 response with o/w squalene. Cytokine production was not significantly stimulated by alum and CpG adjuvants, that in turn downregulated the Th2-biased cytokine response elicited by immunization with H56 antigen alone. Taken together, these results show the fundamental role of the adjuvant in the vaccine formulation to promote a primary CD4^+^ T cell response and the different modulation of the effector response according to the adjuvant used. Therefore, CD4^+^ T cell priming can be considered an important early biomarker of different classes of adjuvant molecules.

### Primary B Cell Response and Early Antibody Secretion

**Figure 4:**
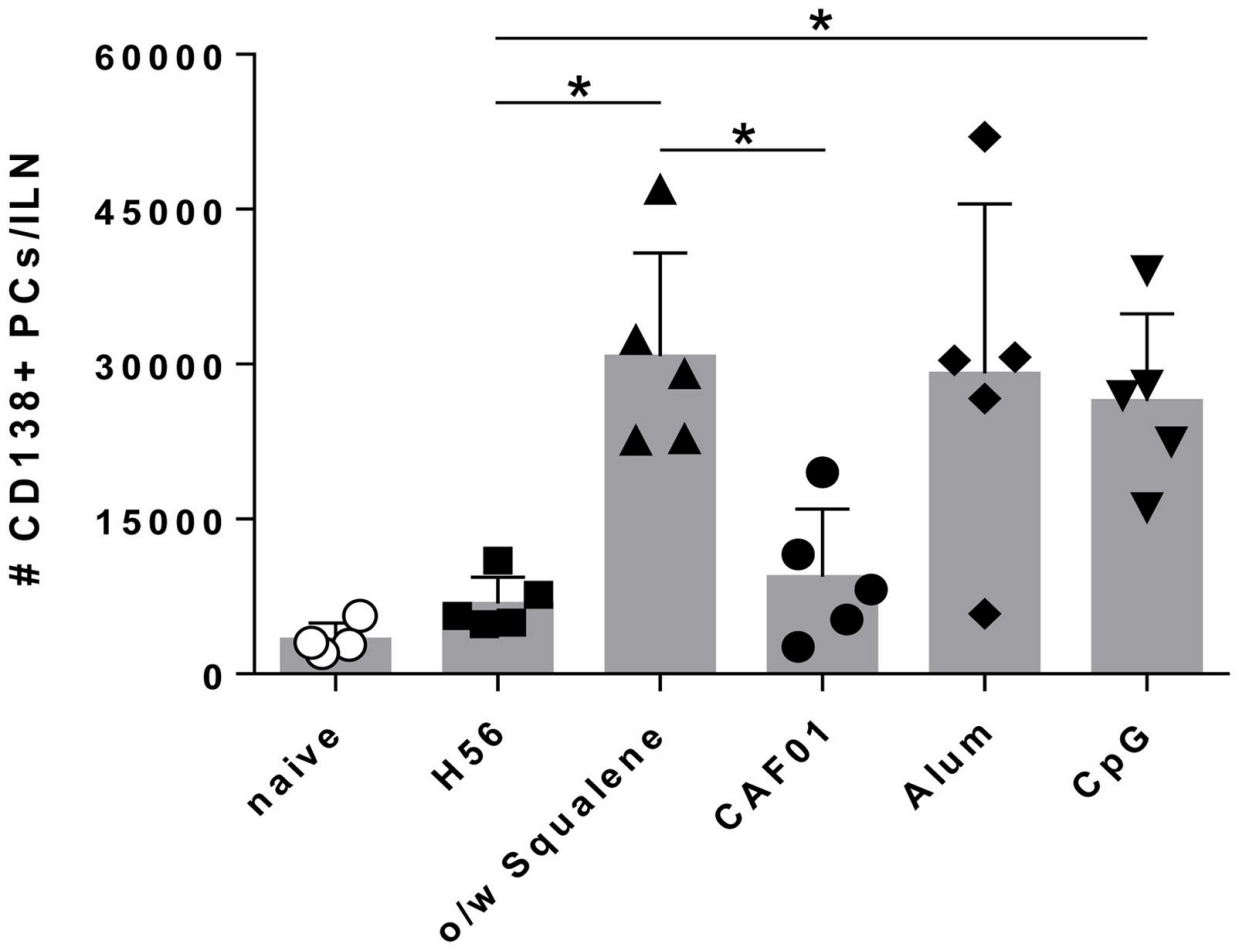
**Plasma cells generation in draining lymph nodes**. C57BL/6 mice were subcutaneously immunized with H56 alone (H56) or combined with different adjuvants (o/w squalene, CAF01, alum, and CpG), and the induction of short-lived plasma cells was analyzed in dLN, 12 days after immunization. Values are reported as number of CD3^−^ IgD^−^ CD138^+^ B220^int^ plasma cells detected in the organ. Bars indicate the mean ± SD of five mice per group; data are representative of two independent experiments. Mann–Whitney test with Bonferroni correction for multiple pairwise comparisons was used for assessing statistical difference between each group immunized with adjuvant and the H56-immunized group. Kruskal–Wallis test, followed by Dunn’s post test for multiple comparisons, was used to assess the statistical difference between groups immunized with different adjuvants (*P ≤ 0.05).

In the extra-follicular reaction, some antigen-primed B cells differentiate in short-lived PCs producing low affinity antibodies, detectable in serum at low concentration a few days after immunization (16, 18). Therefore we assessed the induction of short-lived PCs within draining lymph nodes and quantified the amount of H56-specific IgG and IgG subclasses, in order to define the influence of different adjuvants on the induction of the early primary humoral response. Twelve days after priming, the development of IgD^−^ CD138^+^ B220^int^ short-lived PCs was detected in lymph nodes of mice immunized with H56 combined with o/w squalene, alum, or CpG (*P* ≤ 0.05 versus mice immunized with H56 alone), while the presence of CAF01 did not increase the amount of short-lived PCs (Figure 4).

**Figure 5:**
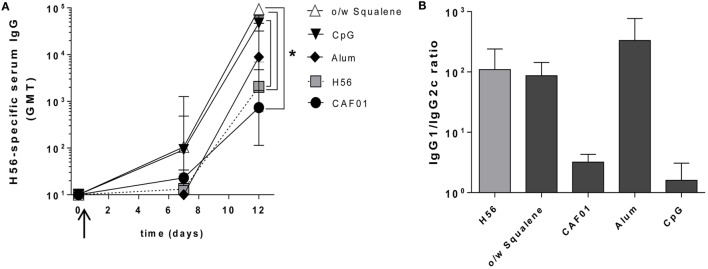
**Early Antigen-specific IgG response**. C57BL/6 mice were subcutaneously immunized with H56 alone (H56) or combined with different adjuvants (o/w squalene, CAF01, alum, and CpG), and humoral response was analyzed in blood **(A)** H56-specific IgG serum response detected by ELISA on sera collected 7 and 12 days after SC immunization. Antibody titers were expressed as the reciprocal of the highest dilution with an OD value ≥0.2 after background subtraction. Values are reported as GMT ± SD of five to eight mice per group, from two independent experiments. Mann–Whitney test, with Bonferroni correction for multiple pairwise comparisons, was used for assessing the statistical difference between each group immunized with adjuvant and the H56-immunized group. Kruskal–Wallis test, followed by Dunn’s post test for multiple comparisons, was used to assess the statistical difference between groups immunized with different adjuvants (*P ≤ 0.05). **(B)** Ratio of H56-specific IgG1 and IgG2c subclasses assessed in serum of each animal. Data are reported as mean ± SEM for each group.

A week after priming, the early H56-specific serum IgG response was elicited by formulations containing o/w squalene or CpG (GMT 100), but not with CAF01, alum, or antigen alone (GMT <20) (Figure 5A). A further and consistent increase was detected at day 12, especially in groups immunized with H56 combined with o/w squalene (GMT 95,000) and CpG (GMT 47,000) with respect to mice immunized with H56 antigen alone (GMT 2,030, *P* ≤ 0.05). The presence of alum did not significantly increase the antibody response (GMT 8,900) compared to antigen alone, while the response was very low with CAF01 (GMT 740) (Figure 5A).

Immunization with H56 alone or combined with o/w squalene or alum elicited a prevalence of IgG1 subclass, indicative of a Th2-biased response, while the IgG1/IgG2c ratio observed with both CpG and CAF01 clearly indicated the strong induction of IgG2c subclass, indicative of a Th1 cell profile (Figure 5B).

The analysis of the early antibody response, which correlates with the induction of short-lived PCs observed in lymph nodes (Figure 4), reflects the different modes of action of the analyzed molecules. Compounds, such as o/w squalene and CpG, are extremely rapid in the stimulation of the early humoral response, probably due to a rapid contact between the antigen and the immune system, while alum and CAF01, both known to entrap the antigen slowing down its release, induce a weaker early humoral response, 12 days after priming.

### The Germinal Center Reaction

**Figure 6:**
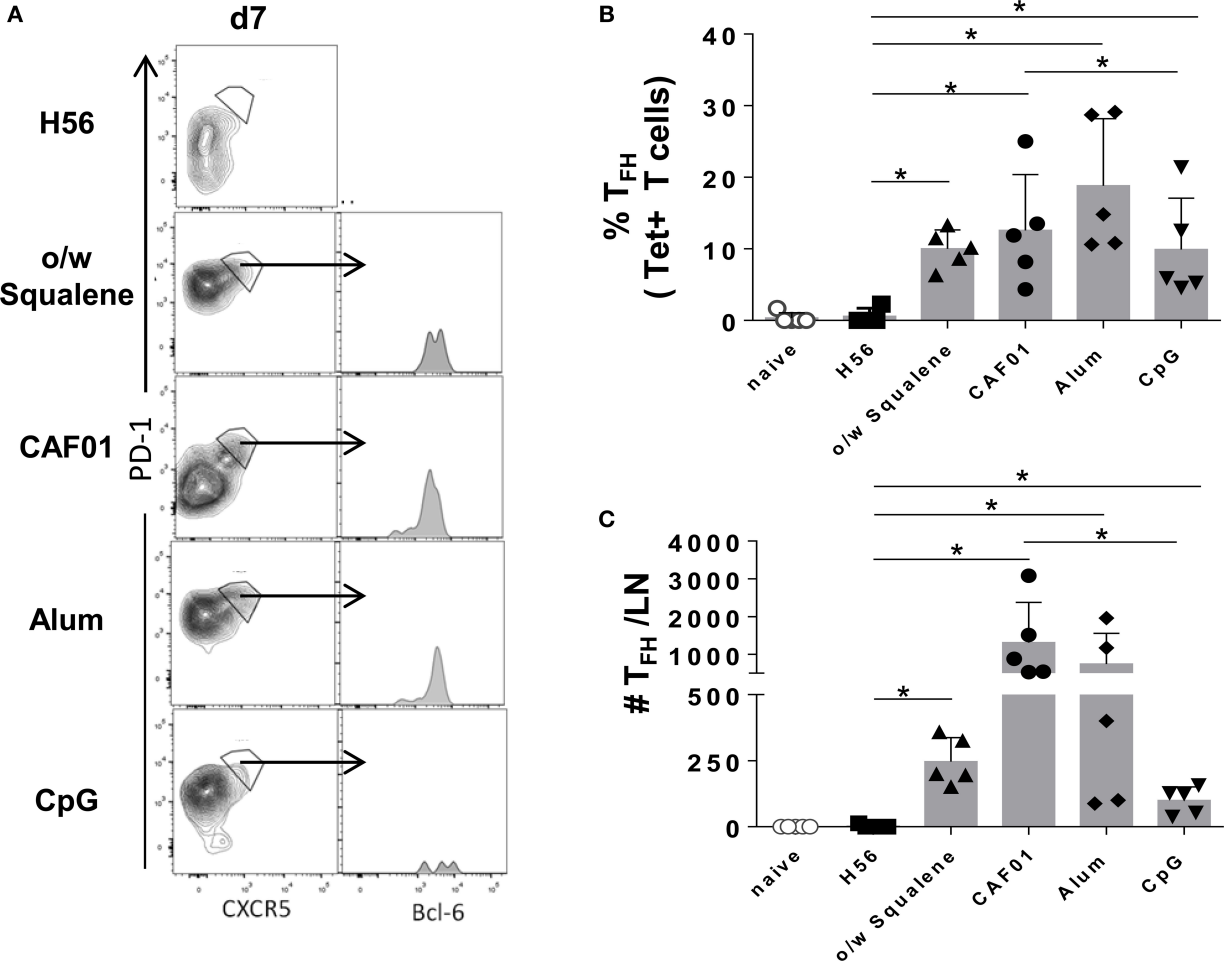
**Tfh cells induction**. Mice were subcutaneously immunized with H56 alone (H56) or combined with different adjuvants (o/w squalene, CAF01, alum, and CpG), and draining lymph nodes were collected 7 days after immunization. **(A)** Analysis of the expression of CXCR5 and PD-1 among tetramer-binding CD4^+^ T cells, for identifying Tfh cells. The intracellular expression of Bcl-6, within CXCR5^+^ PD-1^+^ cells, is reported as histogram. Data are shown from a single animal representative of the group. **(B,C)** Frequencies of Tfh cells, with respect to tetramer^+^ CD4^+^ T cells **(B)** and absolute numbers of Tfh cells per dLN **(C)**, elicited by different vaccine formulations reported as mean ± SD of five mice per group; data are representative of two independent experiments. Mann–Whitney test, with Bonferroni correction for multiple pairwise comparisons, was used for assessing the statistical difference between each group immunized with adjuvant and the H56-immunized group. Kruskal–Wallis test, followed by Dunn’s post test for multiple comparisons, was used to assess the statistical difference between groups immunized with different adjuvants (*P ≤ 0.05).

**Figure 7:**
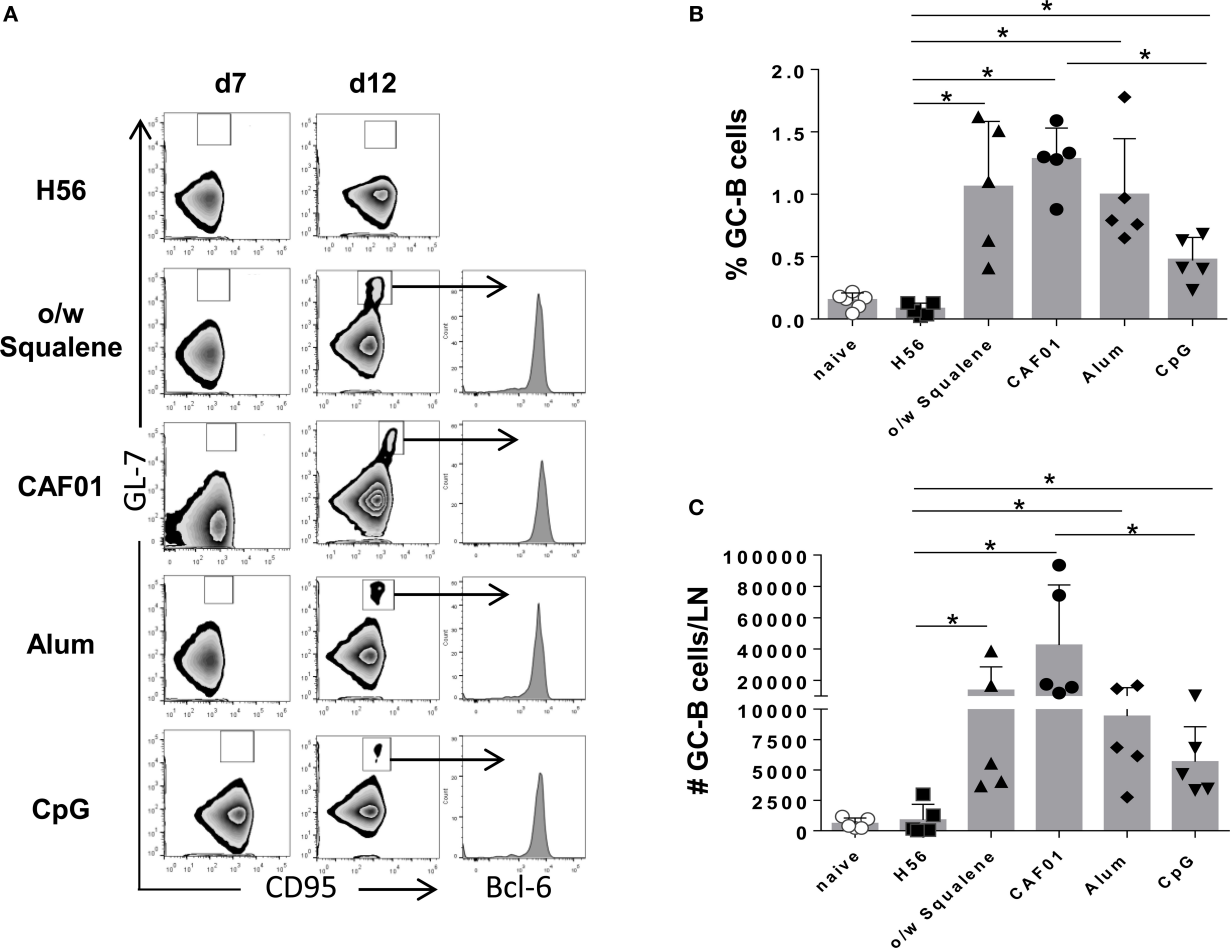
**Germinal center B cells induction**. Mice were subcutaneously immunized with H56 alone (H56) or combined with different adjuvants (o/w squalene, CAF01, alum, and CpG), and draining lymph nodes were collected 7 and 12 days after immunization. **(A)** Germinal center B cells, identified as GL-7^+^ CD95^+^ among B220^+^ B cells in iliac lymph nodes 7 and 12 days after immunization. Intracellular Bcl-6 expression within germinal center B cells was analyzed and reported as histogram. Data are shown from a single animal representative of the group. **(B,C)** Frequencies of GC-B cells, with respect to B220+ B cells **(B)** and absolute numbers of GC-B cells per dLN **(C)** reported as mean ± SD of five mice per group. Mann–Whitney test, with Bonferroni correction for multiple pairwise comparisons, was used for assessing the statistical difference between each group immunized with adjuvant and the H56-immunized group. Kruskal–Wallis test, followed by Dunn’s post test for multiple comparisons, was used to assess the statistical difference between groups immunized with different adjuvants (*P ≤ 0.05).

The induction of the GC reaction is considered an important biomarker of humoral memory response (39), and Tfh cells have emerged as critical for GC formation (17, 40) and for the subsequent generation of plasma cells and memory B cells (16). Tfh and GC-B cell responses were therefore analyzed after immunization with the different vaccine formulations by assessing the expression of functional markers on cells collected from draining lymph nodes. The amount of tetramer-binding CD4^+^ T cells expressing PD-1^high^ CXCR5^high^ Bcl-6^high^ (Tfh) cells was determined 7 days after primary immunization (Figure 6), while B220^+^ GL-7^+^ CD95^+^ GC-B cells were assessed 7 and 12 days after immunization (Figure 7). The intracellular expression of Bcl-6, that is a master regulator for both the GC B cell program (41) and Tfh cell lineage (42), was evaluated in both cell populations. A significant amount of Tfh cells was observed in all vaccine formulations containing adjuvants compared to H56 alone, 7 days after priming (Figure 6). The amount of Ag-specific Tfh cells induced by CpG was much lower compared to the other vaccine formulations, especially compared to CAF01 (Figure 6C; *P* ≤ 0.05). At the same time point, the GC-B cell response was still not induced (Figure 7A), but at day 12, GC-B cells were significantly elicited by formulations containing adjuvants compared to antigen alone (Figures 7B,C). A significant difference in terms of GC-B cells was observed between CAF01 and CpG adjuvants (Figure 7C).

Altogether, these data show that the presence of adjuvants in the vaccine formulation is crucial for the induction of the GC reaction. Nevertheless, the magnitude of the response is deeply influenced by the adjuvant used.

## Discussion

This study focuses on the characterization of primary T and B-cell immune responses upon parenteral priming with different adjuvants combined with the same vaccine antigen. Adjuvants used included a squalene-based oil-in-water emulsion, the liposome system CAF01, aluminum hydroxide, and CpG ODN 1826. By profiling the response after primary immunization, it was possible to highlight the different priming properties of the tested adjuvants (Table 2).

**Table 2:** **Evaluation of early adjuvant activity after primary immunization**.^a^

Adjuvants	Immune response							
	
	T cell response^b^					B cell response^c^		
	
	Ag-specific CD4^+^ T cell response	Th subtype			Ag-specific Tfh cells	Early Ag-specific Ab response	Short-lived PCs	GC-B cells
		Th1	Th2	Th17				
o/w squalene	++	+	+	−	+	+++	++	++
Alum	−	−	−	−	+++	−	+	++
CAF01	+++	+++	−	+++	+++	−	−	+++
CpG	−	−	−	−	+	+++	+	+

*^a^Evaluation of the adjuvant activity: +++, excellent; ++, very good; +, good; –, no response*.

*^b^T cell response assessed 7 days after priming, in draining lymph nodes (Ag-specific CD4^+^ T cell response and Ag-specific Tfh cells) and in the spleen (Th subtype). Ag-specific Th cells were detected with Ag85B-complexed MHC II tetramers; Th subtype was based on Ag-specific cytokine production*.

*^c^B cell response assessed 12 days after priming, in blood (early Ag-specific Ab response) and draining lymph nodes (short-lived PCs and GC-B cells); PCs, plasma cells; GC, germinal center*.

A clear increase of antigen-specific CD4^+^ T cells within draining lymph nodes was observed following primary immunization with formulations containing CAF01 and o/w squalene adjuvants, but not alum and CpG (Table 2). The cytokine profile showed a different effector function of antigen-specific CD4^+^ T-cells induced by different adjuvant formulations. CAF01 was the strongest adjuvant capable of stimulating cytokine secretion, with a well-defined Th1 and Th17 profile, as previously observed following repeated immunizations (14, 31, 43). O/w squalene elicited the release of TNF-α, IFN-γ, and IL-4/IL-13 indicative of a mixed Th1/Th2 response (Table 2). This is in line with the response observed following repeated immunization with the squalene-based o/w emulsions MF59^®^, which primarily induces a humoral response with a mixed Th1/Th2 skewing of T helper cells (14, 44). H56 antigen alone stimulated a Th2-biased cytokine profile, which was down-modulated by the addition of the tested adjuvants. While CAF01 primed for an enhanced antigen-specific cellular immune response, o/w squalene and CpG elicited a rapid and significant humoral immune response. Indeed, o/w squalene and CpG stimulated the induction of short-lived PCs within draining lymph nodes, which correlated with early H56-specific serum IgG antibodies, 12 days after priming (Table 2). On the contrary, immunization with antigen alone or mixed with CAF01 was much less efficient in generating the B cell response at this early time point as observed both in terms of PCs and antibodies. However, high titers (GMT 20,000) have been reached 4 weeks after primary immunization with CAF01 (unpublished data), thus suggesting a slower induction of humoral response, or the stimulation of a late antibody response, probably mediated by the GC-B cells that have been clearly observed with CAF01.

In a vaccination perspective, it is important to elicit the GC reaction, with the follicle-localized GC-B cells capable of generating high-affinity antibody-secreting PCs and memory B cells (39), both critical for immune protection and recall responses. Tested adjuvants, but not antigen alone, promoted the GC reaction with different magnitude. CAF01 elicited the strongest induction of Ag-specific Tfh (PD-1^+^ CXCR5^+^ Bcl-6^+^) and GC-B (B220^+^ GL-7^+^ CD95^+^) cells, which most likely explains the delayed humoral response observed with this adjuvant (14, 45). Also o/w squalene and alum promoted the GC reaction, while CpG induced a mild reaction (Table 2). However, CpG induced a rapid and strong humoral response, in line with what described since the first studies on the adjuvanticity of this compound (46). The observed humoral response elicited by CpG in the absence of a significant primary CD4^+^ T cell response suggests a possible T-independent activation of the B cell response. This could be due to the direct stimulation of TLRs constitutively expressed on naïve B cells in mice (47). In conditions of limited B cell receptor stimulation or T-cell help, TLRs might provide an additional stimulus to responding B cells that also regulates isotype switch (47).

Taken together, the analysis of the early adaptive immune response allows profiling the features of an adjuvant at the beginning of the vaccine-specific immune response. Of course, these data obtained by a single immunization – not followed by a boost – do not allow comparison of adjuvant efficacy in a vaccine perspective but rather show that different adjuvants yield different responses that can be profiled early after primary immunization. This profiling can be instrumental for the rational design of advanced prime–boost immunization for the development of future vaccines. Indeed, understanding the modes of action of adjuvant molecules will allow the development of strategies of vaccination based on a rational design of heterologous prime–boost formulations, or on combinations of adjuvants. Specific adjuvants can be selected for their priming properties, and strategically boosted with other vaccine formulations, in order to optimize the vaccine antigen-specific immune response. The present analysis clearly shows the possibility of identifying the key properties of adjuvants since the first immunization based on serology and cellular immunology approaches. This offers the unique advantage of selecting the optimal priming strategy based on the immunological signature of a vaccine formulation that can be rationally combined with heterologous booster approaches.

## Author Contributions

AC, EP, GPo, and DM conceived and designed the experiments and analyzed the data; AC, EP, FF, and GPa performed the experiments; AC and EP wrote the paper; PA provided reagents; GPo, DM, and PA critically revised the manuscript. All authors read and approved the final manuscript.

## Conflict of Interest Statement

The authors declare that the research was conducted in the absence of any commercial or financial relationships that could be construed as a potential conflict of interest.
