# Supplementary material for: Modulation of Primary Immune Response by Different Vaccine Adjuvants
Source: Front Immunol. 2016 Oct 17;7:427. doi: 10.3389/fimmu.2016.00427 (PMC5066114; doi:10.3389/fimmu.2016.00427)
Supplement: Supplementary file 1 [file Table_1.pdf]

Table S1. Cytokine/chemokine concentration in splenocyte supernatants [a].

**Cytokine/ chemokine**	**H56**	**o/w squalene**	**CAF01**	**alum**	**CpG**
**Eotaxin**	358±96	101 ± 106	516 ± 128	370 ± 147	204 ± 191
**G-CSF**	17,6±36	6,8 ± 84,44	5,4 ± 4,9	12,6 ± 21,9	11 ± 6,9
**GM-CSF**	115±50,5	68,2 ± 44,3	1431 ± 531	147 ± 62,4	37,4 ± 19,3
**IFN-γ**	45±29,8	89,6 ± 98,1	7810 ± 3674	126 ± 48,3	69,4 ± 41,8
**IL-10**	98,2±31,1	19,6 ± 16,2	74,6 ± 22,5	56,4 ± 46,9	27,8 ± 14,8
**IL-12p40**	1,4±2,1	5.9 ± 4,9	51,2 ± 24,2	3,4 ± 3,2	21,2 ± 6,3
**IL-12p70**	34±5	23 ± 24,1	99,6 ± 17,5	29 ± 11,2	21,8 ± 6,9
**IL-13**	1809±483	186 ± 127,6	1556 ± 872	782 ± 535	42 ± 18,5
**IL-17A**	5,6±2,1	16,2 ± 13,4	3779 ± 4416	12,4 ± 16,3	10,4 ± 5,8
**IL-1α**	3±0,7	2,4 ± 3,4	7,6 ± 1,1	2,4 ± 1,1	2 ± 0,7
**IL-1b**	71,4±17,2	32,6 ± 35,1	69,2 ± 34,4	54,8 ± 20,3	55,8 ± 15,8
**IL-2**	587±173	643 ± 127	1324 ± 646	398 ± 267	196 ± 82
**IL-3**	96,8±30	45,8 ± 24,1	364 ± 143	106 ± 60	13,2 ± 6,3
**IL-4**	81,8±12,5	34,8 ± 28,2	26,2 ± 8,5	52 ± 41,3	30,4 ± 13,7
**IL-5**	981±222	114 ± 65	66 ± 44	177 ± 140	7,6 ± 9,1
**IL-6**	21,4±9,9	23,2 ± 27,29	128 ± 35,8	23,4 ± 10,1	4,4 ± 1,9
**KC**	8,8±17,8	3,2 ± 55,6	11,2 ± 5	2,8 ± 14,2	4,6 ± 7,3
**MCP-1**	3608±419	1473 ± 1074	-668 ± 472	3278 ± 1621	-89 ± 210
**MIP-1α**	626±168	140 ± 186	572 ±685	364 ± 170	259 ± 111
**MIP-1β**	2210±487	425 ± 203	800 ±622	1559 ± 704	334 ± 127
**RANTES**	63,6±34,7	60,4 ± 89,7	266 ±41	40 ± 36,4	48 ± 20,3
**TNF-α**	13,2±3,2	9,7 ± 9	21 ±7,2	7,8± 6,9	7,7 ± 1,4

[a] Multiplex immunoassay in culture supernatants of splenocytes from mice naïve or immunized with vaccine formulations including H56 antigen alone or combined with o/w squalene, CAF01, alum, CpG, and restimulated with H56 antigen for 72 hours. Values, expressed as pg/ml, are reported as the arithmetic mean ± SD of H56-stimulated *minus* the respective unstimulated samples, of 5 animal per group.
